# Supplementary material for: Persistently High Procalcitonin and C-Reactive Protein Are Good Predictors of Infection in Acute Necrotizing Pancreatitis: A Systematic Review and Meta-Analysis
Source: Int J Mol Sci. 2024 Jan 20;25(2):1273. doi: 10.3390/ijms25021273 (PMC10816999; doi:10.3390/ijms25021273)
Supplement: Supplementary file 1 [file ijms-25-01273-s001.zip › ijms-2742568-supplementary.pdf]

Title

Persistently High Procalcitonin and C-Reactive Protein are Good Predictors of Infection in Acute Necrotizing Pancreatitis: A Systematic Review and Meta-Analysis

Authors

Dorottya Tarján[1,2,3], Eszter Szalai[1,4], Mónika Lipp[1,2], Máté Verbói[3], Tamás Kói[1,5], Bálint Erőss[1,2,3], Brigitta Teutsch[1,3,6], Nándor Faluhelyi[1,7], Péter Hegyi[1,2,3,8,†,*], Alexandra Mikó[1,3,8,9,†]

Affiliations:

[1]Centre for Translational Medicine, Semmelweis University, Budapest, Hungary, tarjan.dorottya@semmelweis.hu

[2]Institute of Pancreatic Diseases, Semmelweis University, Budapest, Hungary, lipp.monika@semmelweis.hu, eross.balint@semmelweis.hu, hegyi2009@gmail.com

[3]Institute for Translational Medicine, Medical School, University of Pécs, mate.verboi@stud.semmelweis.hu

[4]Department of Restorative Dentistry and Endodontics, Semmelweis University, Budapest, Hungary, szalai.eszter@semmelweis.hu

[5]Department of Stochastics, Institute of Mathematics, Budapest University of Technology and Economics, Budapest, Hungary, koi.tamas@semmelweis.hu

[6]Department of Radiology, Medical Imaging Centre, Semmelweis University, Budapest, Hungary, teutsch.brigitta@semmelweis.hu

[7]Division of Medical Imaging, Medical School, University of Pécs, Pécs, Hungary, faluhelyi.nandor@pte.hu

[8]Translational Pancreatology Research Group, Interdisciplinary Centre of Excellence for Research Development and Innovation University of Szeged, Szeged, Hungary, hegyi2009@gmail.com

[9] Department for Medical Genetics, Medical School, University of Pécs, miko.alexandra@pte.hu

† The last two authors equally contributed.

*Correspondence: hegyi2009@gmail.com; Tel.: +(36-70) 3751031

Corresponding author:

Péter Hegyi MD, PhD, DSc, MAE

Postal address:1038, Tömő utca 25-29, Budapest, Hungary

Tel.: +(36-70) 3751031

E-mail address: hegyi2009@gmail.com

Supplementary Materials

Documentum legends

Document S1: PRISMA checklist

Document S2: Search key

Document S3: Exclusion criteria during the full text selection

Figure and Table Legends

Table S1: Tabular display of risk of bias assessment with QUADAS-2 and QUADAS-C tools

Table S2: Summary of evidence tables (GRADE approach)

Figure S1: ROC plot visualizing the diagnostic performance of PCT and CRP levels

Document S1: PRISMA checklist

Section and Topic	Item #	Checklist item	Location where item is reported
**TITLE**			
Title	1	Identify the report as a systematic review.	1
**ABSTRACT**			
Abstract	2	See the PRISMA 2020 for Abstracts checklist.	4
**INTRODUCTION**			
Rationale	3	Describe the rationale for the review in the context of existing knowledge.	6
Objectives	4	Provide an explicit statement of the objective(s) or question(s) the review addresses.	6
**METHODS**			
Eligibility criteria	5	Specify the inclusion and exclusion criteria for the review and how studies were grouped for the syntheses.	7
Information sources	6	Specify all databases, registers, websites, organisations, reference lists and other sources searched or consulted to identify studies. Specify the date when each source was last searched or consulted.	6
Search strategy	7	Present the full search strategies for all databases, registers and websites, including any filters and limits used.	7
Selection process	8	Specify the methods used to decide whether a study met the inclusion criteria of the review, including how many reviewers screened each record and each report retrieved, whether they worked independently, and if applicable, details of automation tools used in the process.	7
Data collection process	9	Specify the methods used to collect data from reports, including how many reviewers collected data from each report, whether they worked independently, any processes for obtaining or confirming data from study investigators, and if applicable, details of automation tools used in the process.	7
Data items	10a	List and define all outcomes for which data were sought. Specify whether all results that were compatible with each outcome domain in each study were sought (e.g. for all measures, time points, analyses), and if not, the methods used to decide which results to collect.	7
	10b	List and define all other variables for which data were sought (e.g. participant and intervention characteristics, funding sources). Describe any assumptions made about any missing or unclear information.	7
Study risk of bias assessment	11	Specify the methods used to assess risk of bias in the included studies, including details of the tool(s) used, how many reviewers assessed each study and whether they worked independently, and if applicable, details of automation tools used in the process.	7
Effect measures	12	Specify for each outcome the effect measure(s) (e.g. risk ratio, mean difference) used in the synthesis or presentation of results.	8
Synthesis methods	13a	Describe the processes used to decide which studies were eligible for each synthesis (e.g. tabulating the study intervention characteristics and comparing against the planned groups for each synthesis (item #5)).	8
	13b	Describe any methods required to prepare the data for presentation or synthesis, such as handling of missing summary statistics, or data conversions.	8
	13c	Describe any methods used to tabulate or visually display results of individual studies and syntheses.	8
	13d	Describe any methods used to synthesize results and provide a rationale for the choice(s). If meta-analysis was performed, describe the model(s), method(s) to identify the presence and extent of statistical heterogeneity, and software package(s) used.	8
	13e	Describe any methods used to explore possible causes of heterogeneity among study results (e.g. subgroup analysis, meta-regression).	8
	13f	Describe any sensitivity analyses conducted to assess robustness of the synthesized results.	8
Reporting bias assessment	14	Describe any methods used to assess risk of bias due to missing results in a synthesis (arising from reporting biases).	9
Certainty assessment	15	Describe any methods used to assess certainty (or confidence) in the body of evidence for an outcome.	8

Section and Topic	Item #	Checklist item	Location where item is reported
**RESULTS**			
Study selection	16a	Describe the results of the search and selection process, from the number of records identified in the search to the number of studies included in the review, ideally using a flow diagram.	10
	16b	Cite studies that might appear to meet the inclusion criteria, but which were excluded, and explain why they were excluded.	10
Study characteristics	17	Cite each included study and present its characteristics.	10
Risk of bias in studies	18	Present assessments of risk of bias for each included study.	11
Results of individual studies	19	For all outcomes, present, for each study: (a) summary statistics for each group (where appropriate) and (b) an effect estimate and its precision (e.g. confidence/credible interval), ideally using structured tables or plots.	11
Results of syntheses	20a	For each synthesis, briefly summarise the characteristics and risk of bias among contributing studies.	11
	20b	Present results of all statistical syntheses conducted. If meta-analysis was done, present for each the summary estimate and its precision (e.g. confidence/credible interval) and measures of statistical heterogeneity. If comparing groups, describe the direction of the effect.	11
	20c	Present results of all investigations of possible causes of heterogeneity among study results.	11
	20d	Present results of all sensitivity analyses conducted to assess the robustness of the synthesized results.	11
Reporting biases	21	Present assessments of risk of bias due to missing results (arising from reporting biases) for each synthesis assessed.	11
Certainty of evidence	22	Present assessments of certainty (or confidence) in the body of evidence for each outcome assessed.	11
**DISCUSSION**			
Discussion	23a	Provide a general interpretation of the results in the context of other evidence.	12
	23b	Discuss any limitations of the evidence included in the review.	14
	23c	Discuss any limitations of the review processes used.	14
	23d	Discuss implications of the results for practice, policy, and future research.	14
**OTHER INFORMATION**			
Registration and protocol	24a	Provide registration information for the review, including register name and registration number, or state that the review was not registered.	6
	24b	Indicate where the review protocol can be accessed, or state that a protocol was not prepared.	6
	24c	Describe and explain any amendments to information provided at registration or in the protocol.	6
Support	25	Describe sources of financial or non-financial support for the review, and the role of the funders or sponsors in the review.	2
Competing interests	26	Declare any competing interests of review authors.	2
Availability of data, code and other materials	27	Report which of the following are publicly available and where they can be found: template data collection forms; data extracted from included studies; data used for all analyses; analytic code; any other materials used in the review.	2

From: Page MJ, McKenzie JE, Bossuyt PM, Boutron I, Hoffmann TC, Mulrow CD, et al. The PRISMA 2020 statement: an updated guideline for reporting systematic reviews. BMJ 2021;372:n71. doi: 10.1136/bmj.n71

For more information, visit: http://www.prisma-statement.org/

Document S2: Search key

acute AND pancrea* AND necro* AND infect*

Document S3: Exclusion criteria during the full text selection

The studies that compared severe AP and IPN were excluded. [1-107] Moreover, 48 articles were not included in the analysis because they did not contain any relevant data related to laboratory parameters. [61–107].

Table S1: Tabular display of risk of bias assessment with QUADAS-2 and QUADAS-C tools

Study	Test	Risk of bias (QUADAS-2)				Applicability concerns (QUADAS-2)		
		P	I	R	FT	P	I	R
Brand M, 2014		✓	✓	✓	✓	✓	✓	✓
Mándi Y, 2000		✓	✓	✓	✓	✓	✓	✓
Chen HZ, 2017		✓	✓	✓	✓	✓	✓	✓
Wiese ML, 2022		✓	✓	✓	✓	✓	✓	✓
Riché FC, 2003		✓	✓	✓	✓	✓	✓	✓
Ueda T, 2007		✓	✓	?	?	✓	✓	✓
Rotar O, 2022		✓	✓	?	✓	✓	✓	✓
Müller CA, 1999		✓	✓	✓	?	✓	✓	✓
Block S, 1987		?	✓	✓	?	✓	✓	✓
Rotar O, 2019		✓	✓	✓	✓	✓	✓	✓
Dambrauskas Z, 2007		✓	✓	✓	?	✓	✓	✓
Rau B, 2000		✓	✓	✓	?	✓	✓	✓
Zheng L, 2011		✓	✓	✓	?	✓	✓	✓

P = patient selection; I = index test; R = reference standard; FT = flow and timing,
✓ indicates low risk; ✗ indicates high risk; ? indicates unclear risk.

Table S2: Summary of evidence tables (GRADE approach)

a)

Question: Should CRP be used to diagnose INP in NP in the early phase ANP in the early phase ?

Sensitivity	0.45 to 0.65
Specificity	0.73 to 0.89

Outcome	№ of studies (№ of patients)	Study design	Factors that may decrease certainty of evidence					Effect per 1,000 patients tested	Test accuracy CoE
			Risk of bias	Indirectness	Inconsistency	Imprecision	Publication bias	pre-test probability of0%	
**True positives** (patients with INP)	5 studies 204 patients	cross-sectional (cohort type accuracy study)	not serious	not serious	not serious	very serious[a]	none	0 to 0	⊕⊕◯◯ Low
**False negatives** (patients incorrectly classified as not having INP)								0 to 0	
**True negatives** (patients without INP)	5 studies 277 patients	cross-sectional (cohort type accuracy study)	not serious	not serious	not serious	very serious[a]	none	727 to 891	⊕⊕◯◯ Low
**False positives** (patients incorrectly classified as having INP)								109 to 273	

Explanations

a. Confidence intervals are wide

b)

Question: Should PCT be used to diagnose INP in ANP in the early phase ?

Sensitivity	0.61 to 0.78
Specificity	0.58 to 0.75

Outcome	№ of studies (№ of patients)	Study design	Factors that may decrease certainty of evidence					Effect per 1,000 patients tested	Test accuracy CoE
			Risk of bias	Indirectness	Inconsistency	Imprecision	Publication bias	pre-test probability of0%	
**True positives** (patients with INP)	3 studies 120 patients	cross-sectional (cohort type accuracy study)	not serious	not serious	not serious	very serious[a]	none	0 to 0	⊕⊕◯◯ Low
**False negatives** (patients incorrectly classified as not having INP)								0 to 0	
**True negatives** (patients without INP)	3 studies 173 patients	cross-sectional (cohort type accuracy study)	not serious	not serious	not serious	very serious[a]	none	583 to 750	⊕⊕◯◯ Low
**False positives** (patients incorrectly classified as having INP)								250 to 417	

Explanations

a. confidence intervals are wide

c)

Question: Should CRP be used to diagnose INP in ANP in the late phase ?

Sensitivity	0.83 to 0.92
Specificity	0.70 to 0.81

Outcome	№ of studies (№ of patients)	Study design	Factors that may decrease certainty of evidence					Effect per 1,000 patients tested	Test accuracy CoE
			Risk of bias	Indirectness	Inconsistency	Imprecision	Publication bias	pre-test probability of0%	
**True positives** (patients with INP)	3 studies 58 patients	cross-sectional (cohort type accuracy study)	not serious	not serious	not serious	very serious[a]	none	0 to 0	⨁⨁◯◯ Low
**False negatives** (patients incorrectly classified as not having INP)								0 to 0	
**True negatives** (patients without INP)	3 studies 68 patients	cross-sectional (cohort type accuracy study)	not serious	not serious	not serious	very serious[a]	none	700 to 815	⨁⨁◯◯ Low
**False positives** (patients incorrectly classified as having INP)								185 to 300	

Explanations

a. Confidence intervals are wide

d)

Question: Should PCT be used to diagnose INP in ANP in the late phase ?

Sensitivity	0.75 to 0.92
Specificity	0.61 to 0.88

Outcome	№ of studies (№ of patients)	Study design	Factors that may decrease certainty of evidence					Effect per 1,000 patients tested	Test accuracy CoE
			Risk of bias	Indirectness	Inconsistency	Imprecision	Publication bias	pre-test probability of0%	
**True positives** (patients with IPN)	3 studies 95 patients	cross-sectional (cohort type accuracy study)	not serious	not serious	not serious	very serious[a]	none	0 to 0	⨁⨁◯◯ Low
**False negatives** (patients incorrectly classified as not having IPN)								0 to 0	
**True negatives** (patients without IPN)	3 studies 130 patients	cross-sectional (cohort type accuracy study)	not serious	not serious	not serious	very serious[a]	none	612 to 880	⨁⨁◯◯ Low
**False positives** (patients incorrectly classified as having IPN)								120 to 388	

Explanations

a. confidence intervals are wide

e)

Question: Should WBC be used to diagnose INP in ANP in the early phase ?

Sensitivity	0.57 to 0.58
Specificity	0.62 to 0.80

Outcome	№ of studies (№ of patients)	Study design	Factors that may decrease certainty of evidence					Effect per 1,000 patients tested	Test accuracy CoE
			Risk of bias	Indirectness	Inconsistency	Imprecision	Publication bias	pre-test probability of0%	
**True positives** (patients with INP)	3 studies 171 patients	cross-sectional (cohort type accuracy study)	not serious	not serious	not serious	very serious[a]	none	0 to 0	⊕⊕◯◯ Low
**False negatives** (patients incorrectly classified as not having INP)								0 to 0	
**True negatives** (patients without INP)	3 studies 232 patients	cross-sectional (cohort type accuracy study)	not serious	not serious	not serious	very serious[a]	none	620 to 800	⊕⊕◯◯ Low
**False positives** (patients incorrectly classified as having INP)								200 to 380	

Explanations

a. a.Confidence intervals are wide

Figure S1: ROC plot visualizing the diagnostic performance of PCT and CRP levels [28,29,30,31,36].

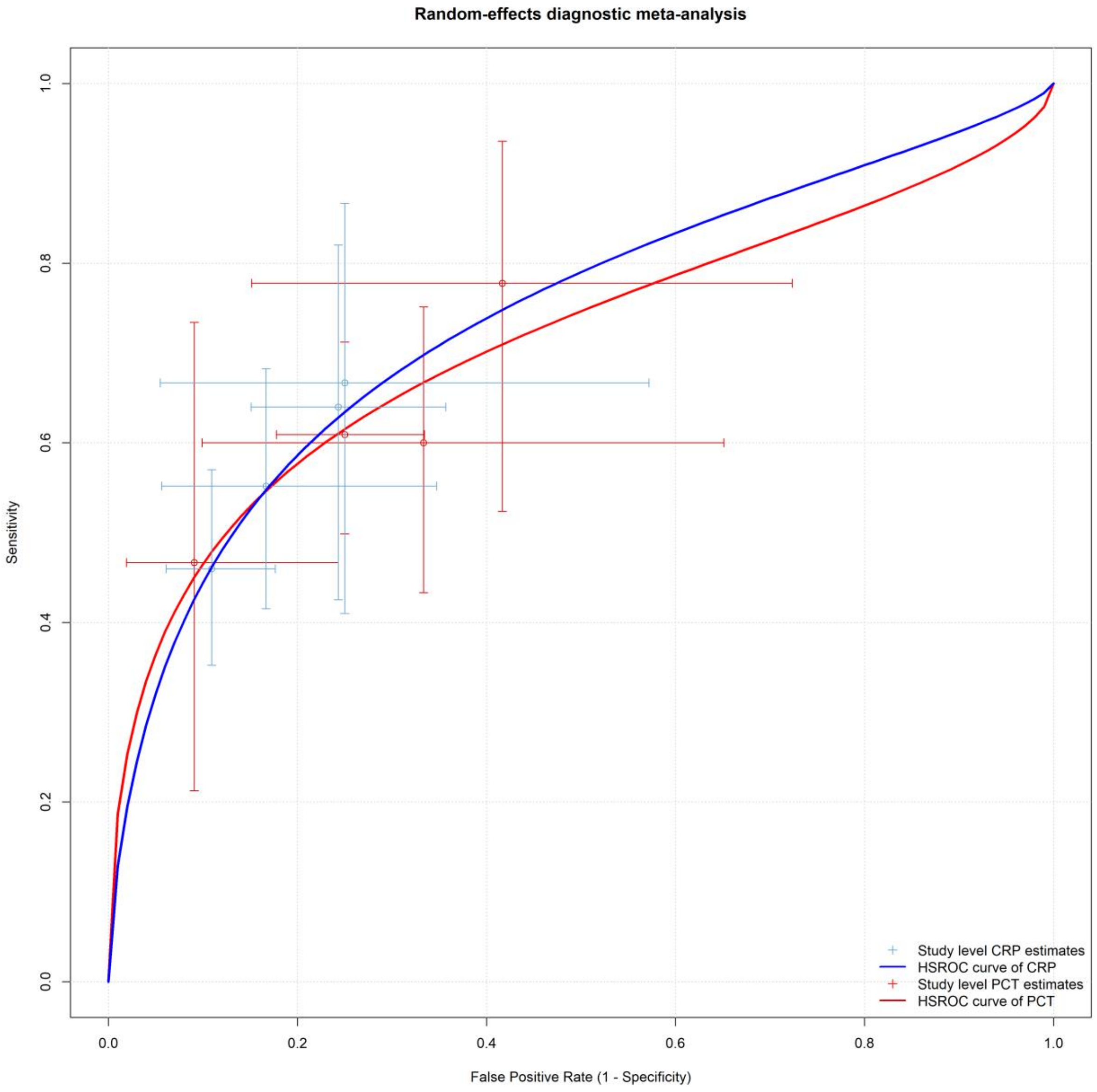

CRP: C-reactive protein

PCT: procalcitonin

Reference

1. Adell Trape M, Alberti P, Dopazo C, Pando E, Vidal L, Hidalgo JN, et al. Are procalcitonin levels on admission related to acute pancreatitis severity and local complications? HPB. 2018;20:S521.
2. Adell Trape M, Hidalgo JN, Pando E, Alberti P, Vidal L, Dopazo C, et al. Serum triglyceride level as a predictor of local complications and severity in acute pancreatitis. HPB. 2018;20:S519.
3. Appasani S, Babu Thandassery R, Basha J, Yadav TD, Attri SV, Singh K, et al. Myriads of markers and scoring systems for multiple events in acute pancreatitis . . . which do we mix and when do we match. Journal of Gastroenterology and Hepatology. 2012;27:374.
4. Armengol-Carrasco M, Oller B, Escudero LE, Roca J, Gener J, Rodríguez N, et al. Specific prognostic factors for secondary pancreatic infection in severe acute pancreatitis. Dig Surg. 1999;16(2):125-9.
5. Bhansali SK, Shah SC, Desai SB, Sunawala JD. Infected necrosis complicating acute pancreatitis: experience with 131 cases. Indian J Gastroenterol. 2003;22(1):7-10.
6. Buddingh KT, Koudstaal LG, van Santvoort HC, Besselink MG, Timmer R, Rosman C, et al. Early angiopoietin-2 levels after onset predict the advent of severe pancreatitis, multiple organ failure, and infectious complications in patients with acute pancreatitis. Journal of the American College of Surgeons. 2014;218(1):26-32.
7. Chooklin S. Prolonged intra-arterial treatment in prophylactic of infected pancreatic necrosis. Surgical Infections. 2013;14(2):A15.
8. Dai P, Qin X, Yan J, Cao F, Gao C, Wang X, et al. Predicting infected pancreatic necrosis based on influential factors among the most common types of acute pancreatitis: a retrospective cohort study. Ann Palliat Med. 2021;10(11):11745-55.
9. De Waele JJ, Blot SI, Vogelaers D, Colardyn F. High infection rates in patients with severe acute necrotizing pancreatitis. Intensive Care Med. 2004;30(6):1248.
10. Deschamps De Boishebert M, Gelsi E, Marine-Barjoan E, Gomercic C, Laveissiere J, Larrey E, et al. Incidence of infections in Necrotizing acute pancreatitis : a french prospective study. Pancreatology. 2020;20:S65.
11. Dhar J, Samanta J, Birda CL, Gupta P, Gupta V, Yadav TD, et al. Dynamics of serum procalcitonin for predicting outcome in patients of infected pancreatic necrosis. United European Gastroenterology Journal. 2021;9(SUPPL 8):740.
12. Dibirov MD, Isaev AI, Dzhadzhiev AB, Ashimova AI, Ataev T. Role of correction of the syndrome of intestinal failure and abdominal hypertension in the prevention of infection of pancreatic necrosis. Khirurgiia. 2016(8):67-72.
13. Ding L, Yu C, Deng F, He WH, Xia L, Zhou M, et al. New Risk Factors for Infected Pancreatic Necrosis Secondary to Severe Acute Pancreatitis: The Role of Initial Contrast-Enhanced Computed Tomography. Dig Dis Sci. 2019;64(2):553-60.
14. Ding L, Zhou YX, He C, Ai JY, Lan GL, Xiong HF, et al. Elevated CA125 levels are associated with adverse clinical outcomes in acute pancreatitis: A propensity score-matched study. Pancreatology. 2020;20(5):789-94.
15. Elavarasan SA, Subramanian S, Karuparthi S, Elangovan B, Gavini S, Sathiyavelavan, et al. Necrotizing pancreatitis our experience from university hospital. HPB. 2012;14:655.
16. Galeev S, Rubtsov M, Abdullaev Y. Factors predicting outcome in severe acute pancreatitis. Pancreatology. 2014;14(3):S62.
17. Galeev SI, Abdullaev YP, Rubtsov MA. Novel model of acute pancreatitis severity and outcome prediction. Pancreas. 2010;39(8):1321.
18. Garret C, Péron M, Le Thuaut A, Le Rhun M, Guitton C, Gournay J, et al. Risks factors and outcomes of infected pancreatic necrosis: Results from a cohort of 148 patients admitted in ICU for severe acute pancreatitis. United European Gastroenterology Journal. 2017;5(5):A199.
19. Hamdi M, Boughariou S, Sfeyhi N, Zbidi B, Zakhama S, Klai F, et al. Procalcitonin and CRP: Predictors of prognosis of severe acute pancreatitis? Anesthesia and Analgesia. 2016;123(3):142.
20. Hrama O, Rotar O, Shafraniuk V, Polyanskyy O. Value of local inflammatory markers for early diagnosis of infected walled-off pancreatic necrosis. Pancreatology. 2019;19:S47-S8.

21. Jakkampudi A, Sarkar P, Koutarapu C, Patil A, Unnisa M, Prasanna A, et al. Alteration of Inflammatory Modulators and Plasma Metabolites in Patients Having Acute Pancreatitis with Infected Pancreatic Necrosis. Pancreas. 2021;50(7):1068.
22. Ji L, Lv JC, Song ZF, Jiang MT, Li L, Sun B. Risk factors of infected pancreatic necrosis secondary to severe acute pancreatitis. Hepatobiliary Pancreat Dis Int. 2016;15(4):428-33.
23. Ke L, Mao W, Li X, Zhou J, Li G, Ye B, et al. The Pancreatitis Activity Scoring System in Predicting Infection of Pancreatic Necrosis. Am J Gastroenterol. 2018;113(9):1393-4.
24. Litvin A, Jarikov O, Filatau A, Litvin V. Clinical decision support system based on an artificial neural network for prediction of infected pancreatic necrosis. Pancreatology. 2015;15(3):S64.
25. Litvin A, Jarikov O, Kovalev V, Khokha V. Clinical decision support system for prediction of infected pancreatic necrosis. Pancreatology. 2010;10(2-3):347-8.
26. Litvin A, Khokha V. The early prediction of infected pancreatic necrosis. Pancreatology. 2009;9(4):498-9.
27. Lytras D, Manes K, Triantopoulou C, Paraskeva C, Delis S, Avgerinos C, et al. Persistent early organ failure: defining the high-risk group of patients with severe acute pancreatitis? Pancreas. 2008;36(3):249-54.
28. Makay R, Issekutz A, Banga P, Belágyi T, Oláh A. [Role of procalcitonin rapid test in the differential diagnosis of uninfected and infected forms of acute pancreatitis]. Magy Seb. 2003;56(1):31-3.
29. Manish K, Siddharth S, Sanjeev S. To study the predictors of infection in asymptomatic patients with walled off necrosis in acute pancreatitis. Journal of Gastroenterology and Hepatology. 2021;36(SUPPL 2):253-4.
30. Oláh A, Belágyi T, Issekutz A, Makay R, Zaborszky A. Value of procalcitonin quick test in the differentiation between sterile and infected forms of acute pancreatitis. Hepatogastroenterology. 2005;52(61):243-5.
31. Rau BM, Kemppainen EA, Gumbs AA, Büchler MW, Wegscheider K, Bassi C, et al. Early assessment of pancreatic infections and overall prognosis in severe acute pancreatitis by procalcitonin (PCT): a prospective international multicenter study. Ann Surg. 2007;245(5):745-54.
32. Rotar O, Khomiak I, Rotar V, Hrama O, Poliansky O. Acute Gastrointestinal Injury Score and its Prognostic Efficacy in Patients with Acute Necrotizing Pancreatitis. HPB. 2021;23:S48.
33. Rotar O, Khomiak I, Rotar V, Khomiak A, Fishbach M, Hrama O. Prognostic efficacy of gastrointestinal injury score at early phase of acute necrotizing pancreatitis. Pancreatology. 2019;19:S6-S7.
34. Rotar O, Khomiak I, Rotar V, Taneja K. Intestinal failure during acute necrotizing pancreatitis. Pancreatology. 2015;15(3):S52.
35. Sah R, Verma GR, Kochhar R, Bhalla A, Banerjee D, Singh R. A prospective study of risk factors predicting infection in acute necrotizing pancreatitis. HPB. 2014;16:642.
36. Shabunin A, Bedin V, Lukin A, Shikov D, Tavobilov M, Grekov D, et al. Management of infected necrotizing pancreatitis. HPB. 2010;12:334-5.
37. Shen D, Ning C, Huang G, Liu Z. Outcomes of infected pancreatic necrosis complicated with duodenal fistula in the era of minimally invasive techniques. Scand J Gastroenterol. 2019;54(6):766-72.
38. Shen D, Wang D, Ning C, Lin C, Cao X, Liu Z, et al. Prognostic factors of critical acute pancreatitis: A prospective cohort study. Dig Liver Dis. 2019;51(11):1580-5.
39. Shen X, Sun J, Ke L, Zou L, Li B, Tong Z, et al. Reduced lymphocyte count as an early marker for predicting infected pancreatic necrosis. BMC Gastroenterol. 2015;15:147.
40. Shinzeki M, Ueda T, Takeyama Y, Yasuda T, Sawa H, Nakajima T, et al. Serum immunosuppressive acidic protein levels in patients with severe acute pancreatitis. Pancreas. 2007;35(4):327-33.
41. Shu W, Wan J, Chen J, He W, Zhu Y, Zeng H, et al. Initially elevated arterial lactate as an independent predictor of poor outcomes in severe acute pancreatitis. BMC Gastroenterol. 2020;20(1):116.
42. Susak YM, Dirda OO, Fedorchuk OG, Tkachenko OA, Skivka LM. Infectious Complications of Acute Pancreatitis Is Associated with Peripheral Blood Phagocyte Functional Exhaustion. Dig Dis Sci. 2021;66(1):121-30.

43. Talamini G, Uomo G, Pezzilli R, Rabitti PG, Billi P, Bassi C, et al. Serum creatinine and chest radiographs in the early assessment of acute pancreatitis. Am J Surg. 1999;177(1):7-14.
44. Tan C, Yang L, Shi F, Hu J, Zhang X, Wang Y, et al. Early Systemic Inflammatory Response Syndrome Duration Predicts Infected Pancreatic Necrosis. J Gastrointest Surg. 2020;24(3):590-7.
45. Talukdar R, Nechutova H, Clemens M, Vege SS. Could rising BUN predict the future development of infected pancreatic necrosis? Pancreatology. 2013;13(4):355-9.
46. Talukdar R, Vege SS, Clemens M. Admission systemic inflammatory response syndrome (SIRS) score predicts the development of primary intra-abdominal infection in patients with acute pancreatitis. Pancreas. 2010;39(8):1351.
47. Thandassery RB, Yadav TD, Dutta U, Appasani S, Singh K, Kochhar R. Hypotension in the first week of acute pancreatitis and APACHE II score predict development of infected pancreatic necrosis. Dig Dis Sci. 2015;60(2):537-42.
48. Thomson JE, Nweke EE, Brand M, Nel M, Candy GP, Fonteh PN. Transient Expression of Interleukin-21 in the Second Hit of Acute Pancreatitis May Potentiate Immune Paresis in Severe Acute Pancreatitis. Pancreas. 2019;48(1):107-12.
49. Tsui NC, Zhao E, Li Z, Miao B, Cui Y, Shen Y, et al. Microbiological findings in secondary infection of severe acute pancreatitis: a retrospective clinical study. Pancreas. 2009;38(5):499-502.
50. Van den Berg F, Van Dalen D, Van Santvoort H, Hyoju S, Besselink M, Zaborina O, et al. Intestinal microbiota and butyrate depletion drive gut-derived infections in necrotizing pancreatitis. HPB. 2020;22:S409.
51. Vasseur P, Devaure I, Sellier J, Delwail A, Chagneau-Derrode C, Charier F, et al. High plasma levels of the pro-inflammatory cytokine IL-22 and the anti-inflammatory cytokines IL-10 and IL-1ra in acute pancreatitis. Pancreatology. 2014;14(6):465-9.
52. Vesentini S, Bassi C, Talamini G, Cavallini G, Campedelli A, Pederzoli P. Prospective comparison of C-reactive protein level, Ranson score and contrast-enhanced computed tomography in the prediction of septic complications of acute pancreatitis. Br J Surg. 1993;80(6):755-7.
53. Vidal L, Pando E, Alberti P, Hidalgo JN, Blanco L, Caralt M, et al. Procalcitonin levels at admission as a predictor of infected pancreatic necrosis in acute pancreatitis. HPB. 2018;20:S520.
54. Wen-Hua H, Nong-Hua L. Establishment of a new multi-factor scoring system for the early prediction of infected pancreatic necrosis. Journal of Gastroenterology and Hepatology. 2017;32:196.
55. Wereszczynska-Siemiatkowska U, Swidnicka-Siergiejko A, Siemiatkowski A, Dabrowski A. Early enteral nutrition is superior to delayed enteral nutrition for the prevention of infected necrosis and mortality in acute pancreatitis. Pancreas. 2013;42(4):640-6.
56. Yasuda H, Suzaki S. Is procalcitonin useful to the diagnosis of infected pancreatic necrosis and pancreatic abscess? Critical Care Medicine. 2011;39:104.
57. Zeng YB, Zhan XB, Guo XR, Zhang HG, Chen Y, Cai QC, et al. Risk factors for pancreatic infection in patients with severe acute pancreatitis: an analysis of 163 cases. J Dig Dis. 2014;15(7):377-85.
58. Zhihua Z, Yixuan D, Yuduo W, Chongchong G, Fei L. Serum CRP, PCT and lipase as predictors of infectious pancreatic necrosis in patients with severe acute pancreatitis. Acta Medica Mediterranea. 2019;35(2):961-5.
59. Zhou J, Chen W, Liu Y, Qu C, Jiang W, Yin J, et al. Trajectories of Lymphocyte Counts in the Early Phase of Acute Pancreatitis Are Associated With Infected Pancreatic Necrosis. Clin Transl Gastroenterol. 2021;12(9):e00405.
60. Zhu Y. Tumor necrosis factor-α and procalcitonin level variations in the serum and their effects on organ function in patients with severe acute pancreatitis during infected stage. Pak J Pharm Sci. 2017;30(4(Suppl.)):1413-6.
61. Alam L, Khan RSA, Kazmi SKH, Din RU. Outcome of patients with acute severe necrotizing pancreatitis in a dedicated hepato-biliary unit of Pakistan. Pak J Med Sci. 2021;37(3):639-45.
62. Bakker OJ, van Santvoort HC, Besselink MGH, Harst E, Hofker HS, Gooszen HG. Prevention, detection, and management of infected necrosis in severe acute pancreatitis. Current Gastroenterology Reports. 2009;11(2):104-10.

63. Baychorov EK, Baturin VA, Gandzha NS, Bairamukov RR, Salpagarov SR, Zinchenko OV, et al. PREDICTION OF THE DEVELOPMENT OF INFECTIOUS COMPLICATIONS WITH PANCRONECROSIS. Medical News of North Caucasus. 2022;17(1):6-9.
64. Beger HG, Bittner R, Block S, Büchler M. Bacterial contamination of pancreatic necrosis. A prospective clinical study. Gastroenterology. 1986;91(2):433-8.
65. Bertsch T, Richter A, Hofheinz H, Böhm C, Hartel M, Aufenanger J. Procalcitonin - A new marker for the acute-phase reaction in acute pancreatitis. Langenbecks Archiv fur Chirurgie. 1997;382(6):367-72.
66. Besselink MG, van Santvoort HC, Boermeester MA, Nieuwenhuijs VB, van Goor H, Dejong CH, et al. Timing and impact of infections in acute pancreatitis. Br J Surg. 2009;96(3):267-73.
67. Büchler MW, Gloor B, Müller CA, Friess H, Seiler CA, Uhl W. Acute necrotizing pancreatitis: Treatment strategy according to th status of infection. Annals of Surgery. 2000;232(5):619-22.
68. Chittajallu V, Vantanasiri K, Simons-Linares CR, Sims A, Christian C, Dirweesh A, et al. Biliary interventions in patients with sterile pancreatic and peripancreatic necrosis increase the risk for infected collection. Pancreas. 2020;49(10):1402.
69. Dai P, Zhang L. Influence of Etiological Factors on Infected Pancreatic Necrosis and Other Local Complications in Acute Pancreatitis. HPB. 2019;21:S407.
70. Ctri. To study the outcomes and natural course of patients with suspected infection of necrosis occurring due to acute pancreatitis disease. http://wwwwhoint/trialsearch/Trial2aspx?TrialID=CTRI/2021/03/031775. 2021.
71. Doctor N, Philip S, Gandhi V, Hussain M, Barreto SG. Analysis of the delayed approach to the management of infected pancreatic necrosis. World J Gastroenterol. 2011;17(3):366-71.
72. Dong X, Mao W, Ke L, Gao L, Zhou J, Ye B, et al. The Diagnosis and Treatment of Local Complications of Acute Necrotizing Pancreatitis in China: A National Survey. Gastroenterol Res Pract. 2021;2021:6611149.
73. Downs-Canner S, Boone B, Steve J, Zureikat A, Lee KK, Zeh HJ, et al. Pancreatic necrosis: A single institutions review of practical adherence to a step-up approach. HPB. 2015;17:57.
74. Dronov O, Kovalska I, Zadorozhna K, Gorlach A, Zemskova M. Impact of obesity on the prognosis of an acute pancreatitis. Pancreatology. 2018;18(4):S10.
75. Fernandez Y Viesca M, Arvanitakis M, Pezzullo M, Jacobs F, Deviere J, Delhaye M. Necrotizing pancreatitis: Can diffusion-weighted magnetic resonance imaging help in determining infection? United European Gastroenterology Journal. 2019;7(8):789.
76. Götzinger P, Wamser P, Barlan M, Sautner T, Jakesz R, Függer R. Candida infection of local necrosis in severe acute pancreatitis is associated with increased mortality. Shock. 2000;14(3):320-3; discussion 3-4.
77. Grendell JH. Persisting early hypotension: is this why necrosis gets infected in acute pancreatitis? Dig Dis Sci. 2015;60(2):285-7.
78. Hilton R, Penn J, Elsaid M, Sarkar A, Pawa S. Descriptive analysis of patients admitted with necrotizing pancreatitis: A tertiary care center experience. American Journal of Gastroenterology. 2016;111:S1227.
79. Horibe M, Sasaki M, Sanui M, Sugiyama D, Iwasaki E, Yamagishi Y, et al. Continuous Regional Arterial Infusion of Protease Inhibitors Has No Efficacy in the Treatment of Severe Acute Pancreatitis: A Retrospective Multicenter Cohort Study. Pancreas. 2017;46(4):510-7.
80. Howard TJ, Patel JB, Zyromski N, Sandrasegaran K, Yu J, Nakeeb A, et al. Declining morbidity and mortality rates in the surgical management of pancreatic necrosis. J Gastrointest Surg. 2007;11(1):43-9.
81. Hu W, Guo Q, Li M, Chen Y. Late infection of pancreatic necrosis: A separate entity in necrotizing pancreatitis with low mortality. European Surgery - Acta Chirurgica Austriaca. 2015;47:S131.
82. Husu HL, Valkonen MM, Leppäniemi AK, Mentula PJ. Occurrence and Risk Factors of Infected Pancreatic Necrosis in Intensive Care Unit-Treated Patients with Necrotizing Severe Acute Pancreatitis. J Gastrointest Surg. 2021;25(9):2289-98.
83. Isenmann R, Rau B, Beger HG. Bacterial infection and extent of necrosis are determinants of organ failure in patients with acute necrotizing pancreatitis. Br J Surg. 1999;86(8):1020-4.

84. Jakkampudi A, Patel A, Chandrakanth K, Unnisa M, Prasanna A, Reddy DN, et al. Identification of molecular characteristics of infected necrosis in patients with acute pancreatitis: Implications for targeted prophylactic interventions. Indian Journal of Gastroenterology. 2022;41(SUPPL 1):S3-S4.
85. Jakkampudi A, Patil A, Panyala B, Sarakar S, Sarkar P, Duvvuru NR, et al. Molecular characteristics of infected pancreatic necrosis in human acute pancreatitis. Journal of Gastroenterology and Hepatology. 2019;34:487.
86. Jakkampudi A, Sarkar P, Sarkar S, Duvvur NR, Unnisa M, Talukdar R. MOLECULAR PHENOTYPING OF INFECTED PANCREATIC NECROSIS (IPN) IN PATIENTS WITH ACUTE PANCREATITIS: POTENTIAL FOR BIOMARKER DEVELOPMENT. Gastroenterology. 2020;158(6):S-327.
87. Kaleem A, Afzal A, Asghar MS, Rafique A, Yousaf S, Khokhar YM. Factors affecting development of infected pancreatic necrosis after severe acute pancreatitis. Rawal Medical Journal. 2022;47(3):569-72.
88. Litvin AA, Garikov OG, Andrews IV. Prediction of infected pancreatic necrosis using an artificial neural network. Surgical Infections. 2009;10(2):227.
89. Lu JD, Cao F, Ding YX, Wu YD, Guo YL, Li F. Timing, distribution, and microbiology of infectious complications after necrotizing pancreatitis. World J Gastroenterol. 2019;25(34):5162-73.
90. Malina P, Cejp V, Jabor A. Interleukin-6 as a necrosis infection marker in severe acute pancreatitis. Clinical Chemistry and Laboratory Medicine. 2011;49:S589.
91. McGuire SP, Keller SL, Maatman TK, Lewellen KA, Ceppa EP, House MG, et al. Obesity Worsens Local and Systemic Complications of Necrotizing Pancreatitis and Prolongs Disease Course. J Gastrointest Surg. 2022;26(10):2128-35.
92. Mikhaĭlusov SV, Moiseenkova EV, Smirnova NA, Bogdanova LS, Vorob'eva EA, Eshtrekov MS. [Laboratory diagnosis of infected pancreonecrosis]. Klin Lab Diagn. 2010(11):3-7.
93. Moran RA, Jalaly NY, Kamal A, Rao S, Klapheke R, James TW, et al. Ileus is a predictor of local infection in patients with acute necrotizing pancreatitis. Pancreatology. 2016;16(6):966-72.
94. Moran RA, Halloran C, Guo Q, Umapathy C, Jalaly NY, Jain S, et al. Early infection is an independent risk factor for increased mortality in patients with culture-confirmed infected pancreatic necrosis. Pancreatology. 2022;22(1):67-73.
95. Moran RA, Yahyapourjalaly N, James T, Rao S, Kamal A, Singh VK. Factors associated with the development of infected pancreatic necrosis: A cohort study from a tertiary referral center. Pancreas. 2015;44(8):1400.
96. Pulay I, Konkoly TM, Arkosy M, Tarjányi M, Flautner L. [Risk factors of infected pancreatic necrosis, its microbiology and antibiotic treatment]. Orv Hetil. 1997;138(18):1113-7.
97. Rau BM, Bothe A, Kron M, Beger HG. Role of early multisystem organ failure as major risk factor for pancreatic infections and death in severe acute pancreatitis. Clin Gastroenterol Hepatol. 2006;4(8):1053-61.
98. Remes-Troche JM, Uscanga LF, Peláez-Luna M, Duarte-Rojo A, González-Balboa P, Teliz MA, et al. When should we be concerned about pancreatic necrosis? Analysis from a single institution in Mexico City. World J Surg. 2006;30(12):2227-33; discussion 34-5.
99. Sonbare DJ. Organ Failure and Infection in Necrotizing Pancreatitis: What Are the Predictors of Mortality? Annals of Surgery. 2017;265(5):e63-e4.
100. Terzin V, Földesi I, Róka R, Szepes Z, Wittmann T, Czakó L. Usefulness of serum calprotectin in the determination of the severity of acute pancreatitis. Pancreatology. 2013;13(3):S35.
101. Thiruvengadam N, Miranda J, Masadeh M, Arain M. The pancreatitis activity scoring system predicts post-intervention outcomes and early readmission in patients with infected pancreatic necrosis. American Journal of Gastroenterology. 2019;114:S49-S50.
102. Thiruvengadam NR, Miranda J, Kim C, Behr S, Arain MA. THE PANCREATITIS ACTIVITY SCORING SYSTEM PREDICTS CLINICAL OUTCOMES IN PATIENTS WITH INFECTED PANCREATIC NECROSIS. Gastroenterology. 2020;158(6):S-1130-S-1.
103. Umapathy C, Raina A, Saligram S, Papachristou GI, Rabinovitz M, Chennat J, et al. Natural history after acute necrotizing pancreatitis (NP): A large U.S. tertiary care experience. Gastroenterology. 2015;148(4):S113.

104. van Grinsven J, van Brunschot S, van Baal MC, Besselink MG, Fockens P, van Goor H, et al. Natural History of Gas Configurations and Encapsulation in Necrotic Collections During Necrotizing Pancreatitis. J Gastrointest Surg. 2018;22(9):1557-64.
105. Wu XM, Ji KQ, Wang HY, Li GF, Zang B, Chen WM. Total enteral nutrition in prevention of pancreatic necrotic infection in severe acute pancreatitis. Pancreas. 2010;39(2):248-51.
106. Yee E, Sood AJ, Maatman TK, Colgate C, Zyromski NJ. 179 CAN BASELINE FRAILTY METRICS PREDICT OUTCOMES IN NECROTIZING PANCREATITIS? Gastroenterology. 2020;158(6):S-1497.
107. Guo Q, Li A, Xia Q, Liu X, Tian B, Mai G, et al. The role of organ failure and infection in necrotizing pancreatitis: a prospective study. Ann Surg. 2014;259(6):1201-7.
